# *In Vitro* Differentiation of Bone Marrow Mesenchymal Stem Cells into Neuron-Like Cells by Cerebrospinal Fluid Improves Motor Function of Middle Cerebral Artery Occlusion Rats

**DOI:** 10.3389/fneur.2016.00183

**Published:** 2016-10-27

**Authors:** Ying Ye, Yi-ran Peng, Shu-qun Hu, Xian-liang Yan, Juan Chen, Tie Xu

**Affiliations:** ^1^Jiangsu Province Key Laboratory of Anesthesiology, Institute of Emergency Rescue Medicine, Xuzhou Medical University, Xuzhou, Jiangsu, China; ^2^Emergency Center, Affiliated Hospital of Xuzhou Medical University, Xuzhou, Jiangsu, China; ^3^Department of Clinical Medicine, Xuzhou Medical University, Xuzhou, China

**Keywords:** cerebrospinal fluid, bone marrow mesenchymal stem cells, BMSC-derived neural cells, middle cerebral artery occlusion

## Abstract

Bone marrow mesenchymal stem cells (BMSCs) represent a promising tool for stem cell-based therapies. However, the majority of BMSC transplants only allow for limited recovery of the lost functions. We previously found that human cerebrospinal fluid (hCSF) is more potent than growth factors in differentiating human BMSCs into neuron-like cells *in vitro*. In this study, we studied the effect of transplantation of rat BMSC-derived neuron-like cells (BMSC-Ns) induced by hCSF into rat brain with middle cerebral artery occlusion (MCAO). The survival and differentiation of the transplanted cells were determined using immunofluorescence staining of bromodeoxyuridine. The recovery of neurological function were observed by the modified neurological severity score (modified NSS) at 4, 15, and 32 days after cell transplantation, HE staining for determination of the infarct volume at day 32 after cell transplantation. Transplantation of BMSC-Ns or BMSCs significantly improved indexes of neurological function and reduced infarct size in rats previously subjected to MCAO compared with those in the control group. Remarkably, 32 days after transplantation, rats treated with BMSC-Ns presented a smaller infarct size, higher number of neuron-specific, enolase-positive, and BrdU-positive cells, and improved neurological function compared with BMSC group. Our results demonstrate that transplantation of hCSF-treated BMSC-Ns significantly improves neurological function and reduces infarct size in rats subjected to MCAO. This study may pave a new avenue for the treatment of MCAO.

## Introduction

Stroke is an important cause of death and disability in adults. However, endogenous neural stem cells (NSCs) in the brain with poor ability to migrate and differentiate into functional neurons only proliferated during a certain period after brain tissue damage (1). Bone marrow mesenchymal stem cells (BMSCs) present a diversity of functions. Transplantation of these cells facilitates recovery in animal models of cerebral ischemia *via* mechanisms that may include replacement of damaged cells, neuroprotection, induction of axonal sprouting, and neovascularization (2–6). However, studies have shown that the ratio of BMSCs differentiating into glial cells and fibroblasts after transplantation *in vivo* is higher than the ratio of BMSCs differentiating into neuron-like cells (7, 8). Excessive glia and fibroblasts can increase glial scar formation (9). Therefore, we theorized that BMSCs *in vitro* differentiation into more neuronal precursor cells than glia and fibroblasts before transplantation may represent a more effective therapeutic strategy. We previously successfully induced the differentiation of BMSCs into neuron-like cells using human cerebrospinal fluid (hCSF) (10, 11). In the present study, human CSF-treated rat BMSCs were injected stereotaxically into the brain of rats with focal cerebral ischemia caused by middle cerebral artery occlusion (MCAO). The survival and differentiation of the implanted cells and recovery of neurological function were determined to explore the feasibility of using hCSF-treated BMSC-derived neuron-like cells (BMSC-Ns) transplantation to treat focal cerebral ischemia.

## Materials and Methods

### Experimental Animals

Three-month-old Sprague-Dawley rats (270–300 g, *n* = 72) and 1-month-old Sprague-Dawley rats (100–150 g, *n* = 16) were provided by the Experimental Center of Xuzhou Medical College. Rats were housed in groups of four per standard polycarbonate cage in a temperature- and humidity-controlled colony room with 12-h/12-h light/dark cycle (lights on at 07:00 a.m.) and *ad libitum* access to food and water. All animal experiments were conducted and approved by the Institutional Animal Care and Use Committee of Xuzhou Medical College and the methods were carried out in accordance with approved guidelines (Assurances No. 2015-46, 2015-47).

### Separation, Cultivation, and Induction of MSCs

The BMSCs were isolated and harvested as previously described (12). In brief, Bone marrow cells from rats weighting 100–150 g were harvested and introduced into T25 flask (BD Falcon™, Franklin lakes, NJ, USA) and cultured in a complete medium containing 10% fetal bovine serum (FBS, HyClone, Logan, UT, USA) and assorted by fluorescence-activated cell sorting (FACS), as described previously (10, 11).

### Bromodeoxyuridine – Labeling

P3-generation of BMSCs were cultured in medium containing 10 μmol/l bromodeoxyuridine (BrdU) solution (Sigma, St. Louis, MO, USA). At 80% confluent, cells were collected and resuspended in PBS. Approximately 2 × 10^3^ cells were plated in a 24-well plate. Rabbit anti-mouse BrdU antibody (1:200; Wuhan Boster Biological Technology, Ltd., China) was added and incubated overnight at 4°C before goat anti-rabbit IgG-HRP polymer (Sigma, St. Louis, MO, USA) was added. Diaminobenzidine was added for color development, hematoxylin for re-staining, and hydrochloric acid in alcohol for differentiation. The cells were then dehydrated using a gradient of ethanol concentrations, cleared with xylene, and mounted with neutral gum. BrdU-labeled cells and total cells were counted under five non-overlapping fields.

### *In Vitro* Differentiation of BMSCs into Neurons

P3-generation BMSCs were incubated with medium containing 20% hCSF. The CSF samples were obtained from healthy adult human volunteers. The medical ethics committee of the affiliated hospital of Xuzhou medical college approved the study (Assurances No. xyfylw2012034). At day 4 post-induction by hCSF, cells were digested with 0.25% trypsin and resuspended to a concentration of 1 × 10^7^ cells/ml. Cell differentiation state was examined using microscopy, Western blot analysis, and fluorescent immunohistochemistry staining as described previously (10, 11).

### Development of Focal Cerebral Ischemia and Neurological Assessment

A rat model of focal cerebral ischemia was generated using suture-occlusion of the middle cerebral artery as reported by Longa et al. (13). Briefly, the right middle cerebral artery was occluded. Twenty-four hours after the occlusion, the rats underwent neurological scoring using the 5-level neurological severity score ranging from 1 to 3 points. Every effort was made to minimize the number of animals used and animal suffering.

Middle cerebral artery occlusion rats were randomly divided into three groups (*n* = 24 rats per group). The BMSC-N group was injected with 20 μl of BrdU-labeled BMSC-Ns, which had been induced with hCSF for 4 days; the BMSC group was injected with 20 μl of BrdU-labeled P3-generation BMSCs; the control group was injected with 20 μl of artificial CSF (Sigma, St. Louis, MO, USA). The cells (1 × 10^6^ cells) were injected into the lateral ventricles stereotaxically 7 days after successful MCAO. Neurological assessment was based on a modified neurological severity score (modified NSS) method (14) (Table S1 in Supplementary Material) on day 7 after MCAO (day 0 before cell transplantation), and days 4, 15, and 32 after cell transplantation.

### Cell Survival and Migration after Transplantation

After being anesthetized with pentobarbital sodium, rats were perfused transcardially with saline, and the brain was removed and frozen immediately in liquid nitrogen. Brain was sliced at 14 μm thickness using a LEICA 1800 cryostat microtome. The slices were stained alternatively for neuronal specific enolase (NSE, neuronal marker) or rabbit anti-rats glial fibrillary acidic protein (GFAP, a astrocyte marker), mouse anti-BrdU antibody (1:100), rabbit anti-rats NSE antibody (1:200; Wuhan Boster Biological Technology, Ltd., China) or rabbit anti-rats GFAP antibody (1:200; Wuhan Boster Biological Technology, Ltd., China) at 37°C. After being washed with PBS, the FITC-labeled anti-mouse IgG and Texas Red-labeled anti-rabbit IgG (Sigma, St. Louis, MO, USA) were added to the tissue drop wise and then incubated in the dark for 60 min before incubation with 4′,6-diamidino-2-phenylindole (DAPI; Sigma, St. Louis, MO, USA) for 5 min to label cell nuclei. Five non-overlapping fields from the cerebral ischemia side were selected for immunofluorescence observation with a total of 20 fields per animal. NSE+ or GFAP+ cells were counted to determine the differentiation rate of BrdU cells into neuron or glia. The differentiation rate was calculated as (BrdU/NSE double-labeled cells or BrdU/GFAP double-labeled cells/total BrdU cells) × 100%.

### Determination of Volume of Cerebral Infarction by HE Staining

Rats were given intravenous injections of 60 mg/kg pentobarbital sodium at day 7 after modeling and 32 days (*n* = 6 in each group) after transplantation. The brain tissues were quickly removed covering the infarct area and the surrounding brain tissues. The specimens were fixed in 4% formaldehyde and then placed in 0.01 mol PBS buffer solution (pH = 7.4). Twelve hours later, the specimens were successively placed in 70% ethanol solution, 80% ethanol solution, 90% ethanol solution, 95% ethanol solution, and 100% ethanol solution in order to be dehydrated, followed by transparency with xylene, and then embedded in paraffin. The brain tissues underwent consecutive paraffin section with the LEICA RM2125 microtome. The thickness of each slice was 4 μm. The slices were conventionally dewaxed with xylene and washed with decreasing concentrations of ethanol until only water was used. They were stained with hematoxylin for 5 min and then washed with tap water. They underwent differentiation with hydrochloric acid ethanol for 30 s and then soaked in tap water for 15 min. They were placed in eosin for 2 min and then underwent conventional dehydration, transparency, and mounting. Six consecutive slices in the infarct zone were taken for photographing with a digital camera. An image processing software (i Motic Med 6.0 digital medical image analysis system) was used to measure the infarct size and to calculate the ratio of infarct area to the area of the injured hemisphere.

### Data Analyses

Data are expressed as the mean ± SD. One-way ANOVA and independent samples Student’s *t*-test were used. The significance level was set at *p* < 0.05. All statistical tests were conducted using the SPSS v13 (IBM, Armonk, NY, USA) software package.

## Results

### Morphological Characterization of Isolated, Cultured, and Induced BMSCs

**Figure 1:**
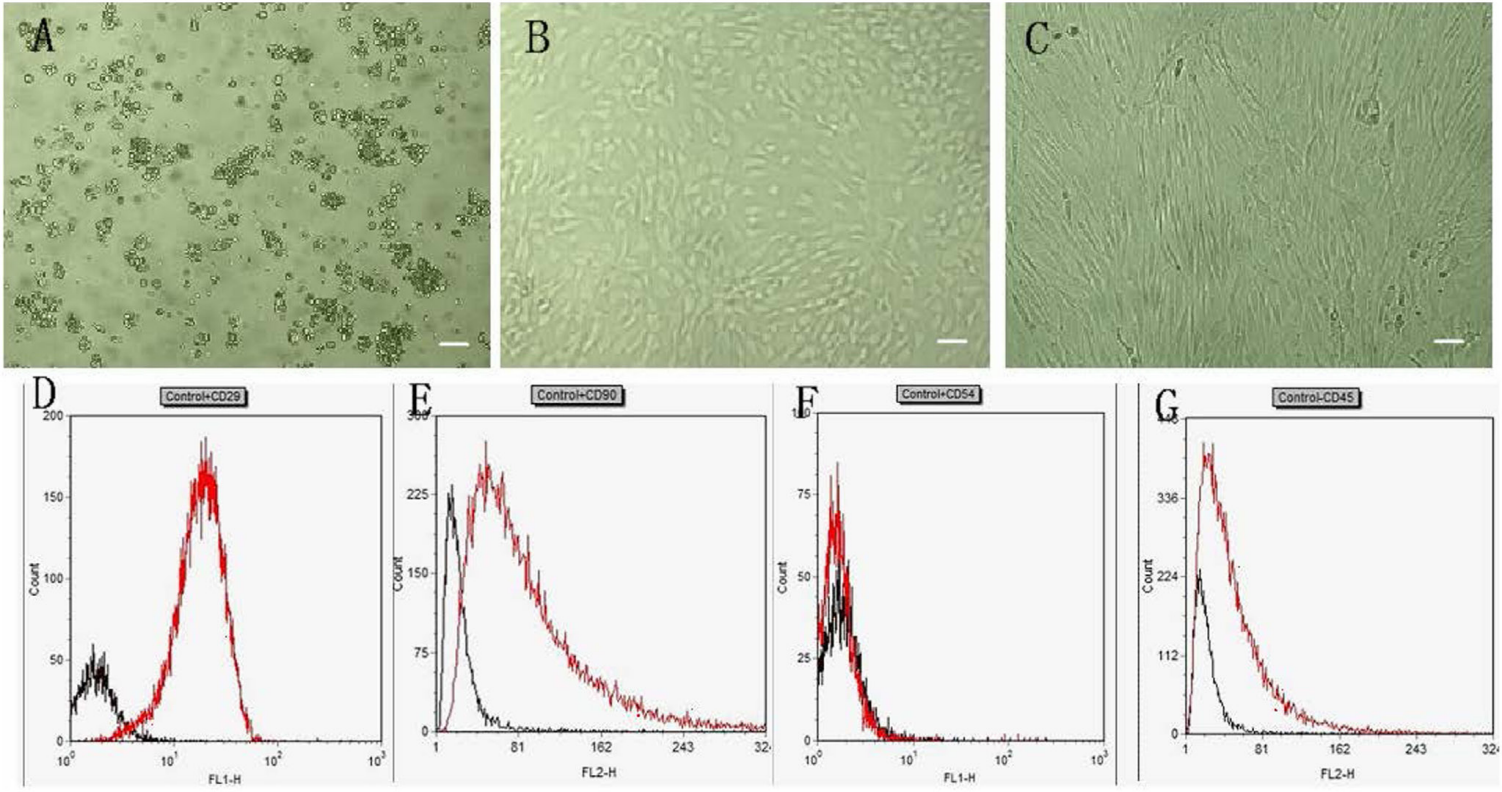
**Characteristics of rat BMSCs**. **(A–C)** show BMSCs cultured for 1, 7 days and the P3-generation cells, respectively (40×). Scale bar = 100 μm. **(D–G)** show flow cytometry analysis of cell surface markers. On P3-generation cells: a approximately 95% of events were positive for CD29 **(D)** and CD90 **(E)** and were negative for CD54 **(F)** and CD45 **(G)**.

**Figure 2:**
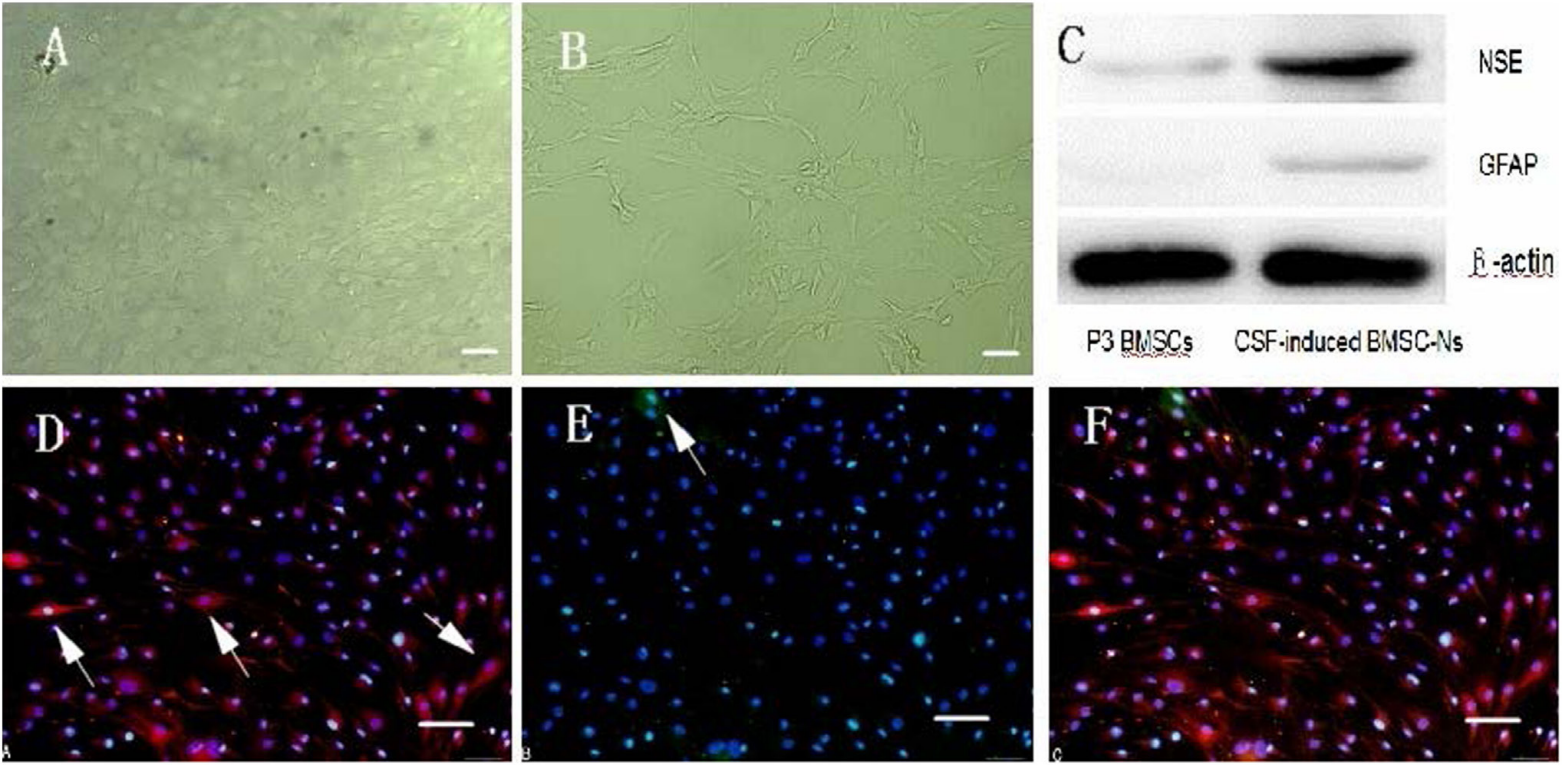
**Characteristics of hCSF-induced rat BMSC-differentiated neuron-like cells**. **(A,B)** show post-induction by hCSF at day 1 and 4 (40×). Scale bar = 100 μm. **(C)** The protein levels were measured by a GELDOC instrument and normalized with respect to β-actin, which was chosen as an internal control. Each experiment was repeated at least three times. Variations in protein expression are given as arbitrary units. The expression of NES in cells induced by hCSF (0.43 ± 0.07) was significantly higher than that of P3-generation BMSCs (0.09 ± 0.03); meanwhile, GFAP also had small amount of expression (0.09 ± 0.02). However, P3-generation of BMSCs almost did not express GFAP (0 ± 0). **(D–F)** show Cells were positive for both NSE and GFAP, but expression levels for NSE were higher than GFAP. Western blot analysis of NSE and GFAP protein expression (200×). Scale bar = 100 μm.

Primary cultured BMSCs appeared round and translucent with good refractivity and had mixed bone marrow mononuclear cells. A small number of adherent cells were visible 24 h after inoculation. These cells were spindle-shaped, similar to fibroblasts, grew in a colony-like manner, and gradually increased in number (Figure 1A). After approximately 1 week, these cells were arranged in a swirled pattern (Figure 1B). The growth rate was elevated with passage (Figure 1C). Thirty-six hours after induction, cell bodies appeared to be retracting, that is, the cells were lighter in color and many protrusions formed below the retracted balls (Figure 2A), which gradually tapered irregularly and then formed into triangles on day 4 (Figure 2B). More than 95% of P3-generation cells were positive for CD29 and CD90 and were negative for CD54 and CD45 (Figures 1D–G). At day 4 post-induction by hCSF, cells were positive for both NSE and GFAP, but expression levels for NSE were higher than GFAP (Figures 2D–F). Western blot analysis of NSE and GFAP protein expression. The protein levels were measured by a GELDOC instrument and normalized with respect to β-actin, which was chosen as an internal control. Each experiment was repeated at least three times. Variations in protein expression are given as arbitrary units. The expression of NES in cells induced by hCSF (0.43 ± 0.07) was significantly higher than that of P3-generation BMSCs (0.09 ± 0.03), meanwhile GFAP also had small amount of expression (0.09 ± 0.02). However, P3-generation of BMSCs almost did not express GFAP (0 ± 0) (Figure 2C).

### Behavioral Assessment

**Figure 3:**
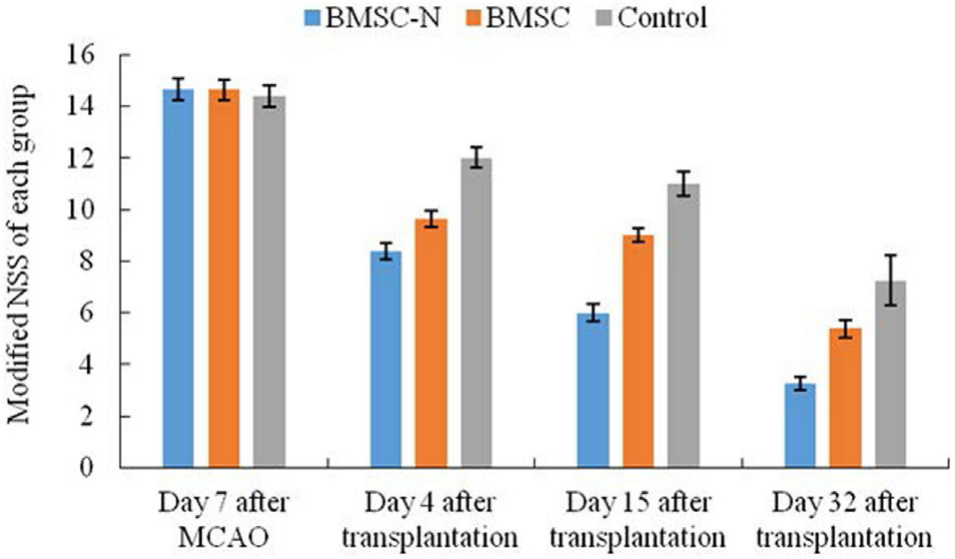
**Modified neurological severity scores (Modified NSS) of each group at day 7 after MCAO, 4, 15, and 32 days after transplantation**. Significance levels are as shown in Table 1.

Middle cerebral artery occlusion rats presented neurological deficits, for example, left limb flexion, circling, falling of the limb to the paralyzed side, as well as other signs. The modified NSS of the rats were not different among the three groups before transplantation. The score of the BMSC-N group were significantly lower than that in the control or the BMSC group (*p* < 0.05). The modified NSS in the BMSC group were significantly lower than those in the control group on days 4 and 15 after transplantation (*p* < 0.05); however, the scores between these groups did not differ significantly on day 32 after transplantation (Figure 3; Table 1).

**Table 1:** **Effects of BMSC-Ns and BMSCs transplantation on neurological performance, BrdU labeling, and cell differentiation (data represent mean ± SD, *n* = 8 per group): modified neurological severity scores with time after transplantation**.

Treatment	Day 7 after MCAO	Day 4 after transplantation	Day 15 after transplantation	Day 32 after transplantation
BMSC-N	14.63 ± 0.42	8.38 ± 0.32^a^^,^^b^^,^^c^	6.00 ± 0.32^a^^,^^b^^,^^c^	3.25 ± 0.25^a^^,^^b^^,^^c^
BMSC	14.63 ± 0.38	9.63 ± 0.32^a^^,^^c^	9.00 ± 0.27^a^^,^^c^	5.38 ± 0.32^c^
Control	14.38 ± 0.42	12.00 ± 0.38	11.00 ± 0.46	7.25 ± 0.96^c^

*^a^Significantly different (p < 0.05) compared with controls during the same time period*.

*^b^Significantly different (p < 0.05) compared with the BMSC group during the same time period*.

*^c^Significantly different (p < 0.05) compared with day 7 after MCAO*.

### BrdU-Positive Cell Labeling after Cell Implantation

**Figure 4:**
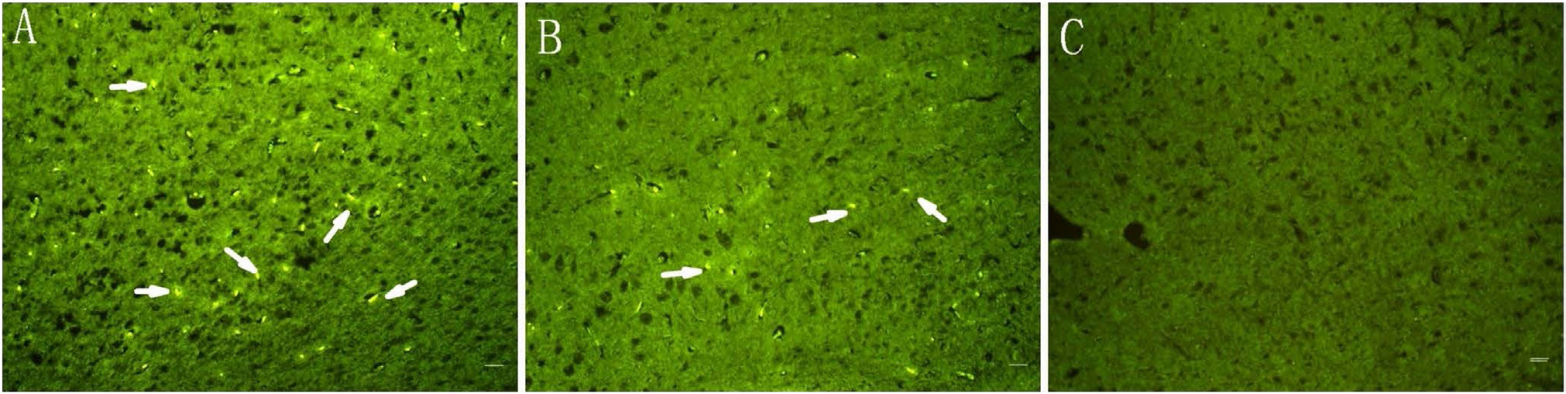
**Immunofluorescent staining of brain slices on day 32 after transplantation**. Cells labeled with green are BrdU-positive cells (arrows). **(A)** BMSC-N group, **(B)** BMSC group, and **(C)** the control group. Scale bar = 100 μm. Significance levels are as shown in Table 2.

BrdU-positive cells were visualized in the BMSC-N and BMSC groups on day 32 after transplantation (Figure 4). These cells were mainly located in the ischemic area and ischemic border zone. Only a few BrdU-positive cells were observed in the contralateral hemisphere. BrdU-positive cells with green labeling were not observed in the control group (Figure 4C). Meanwhile, the density of BrdU-positive cells was significantly higher in the BMSC-N group than that in the BMSC group (Table 2).

**Table 2:** **Effects of BMSC-Ns and BMSCs transplantation on neurological performance, BrdU labeling, and cell differentiation (data represent mean ± SD, *n* = 8 per group): comparison of the number of BrdU-labeled cells**.

Treatment group	BrdU-labeled cells 32 days after transplantation (*N*/5 fields)
BMSC-N	52.31 ± 8.03^a^^,^^b^
BMSC	19.31 ± 2.21^a^
Control	0.00 ± 0.00

*Data represent mean ± SD, with n = 8 per group*.

*^a^Significantly different compared with controls (p < 0.05)*.

*^b^Significantly different compared with the BMSC group (p < 0.05)*.

### BrdU/NSE and BrdU/GFAP Double-Labeling after Cell Transplantation

**Figure 5:**
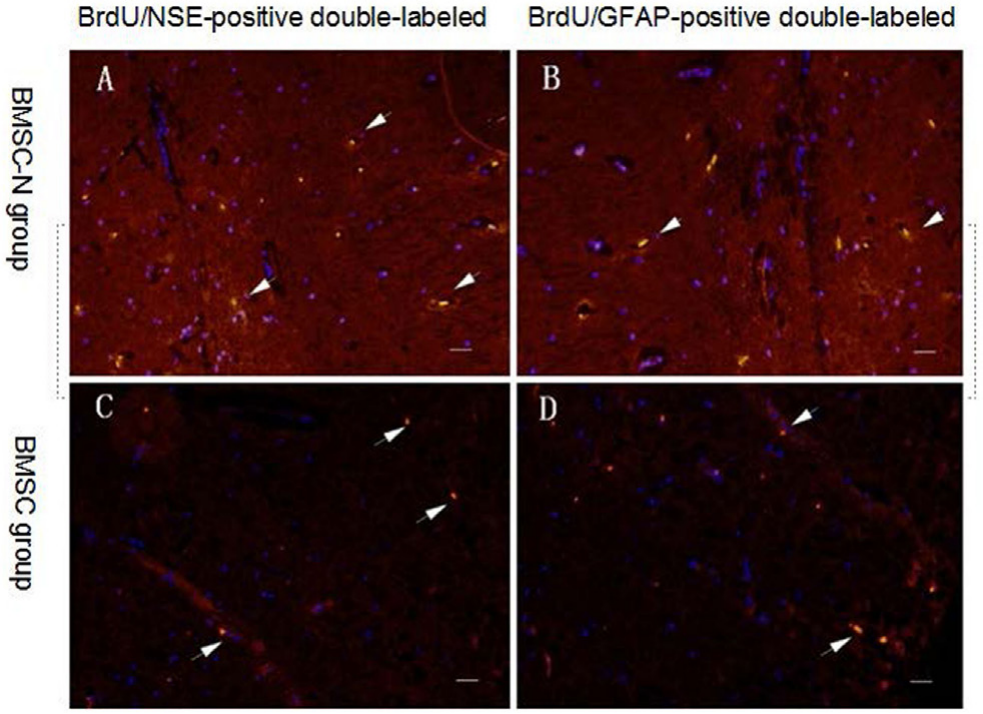
**Distribution of transplanted cells in the ischemic core of the brain on day 32 after transplantation in rats subjected to MCAO**. Green labeling indicates BrdU-positive cells. DAPI (blue) labels nuclei. Immunofluorescent staining showing BrdU-positive transplanted cells (green) and NSE-positive cells (or GFAP-positive cells) (red). BrdU/NSE-positive double-labeled cells (or BrdU/GFAP-positive double-labeled cells) (yellow) **(A,B)** BMSC-N group; **(C,D)** BMSC group. The number of BrdU/GFAP-positive double-labeled cells or BrdU/NSE-positive double-labeled cells in the BMSC-N group was higher than that in the BMSC group. The BMSC-N group showed more BrdU/NSE-positive double-labeled cells than BrdU/GFAP-positive double-labeled cells. Conversely, the BMSC group had more BrdU/GFAP-positive double-labeled cells than BrdU/NSE-positive double-labeled cells. Significance levels are as shown in Table 3. Scale bar = 100 μm.

Both BrdU/GFAP- and BrdU/NSE-positive double-labeled cells were visualized in brain slices from the BMSC-N and BMSC groups on day 32 after cell transplantation. The number of BrdU/GFAP-positive double-labeled cells or BrdU/NSE-positive double-labeled cells in the BMSC-N group was higher than that in the BMSC group. The BMSC-N group showed more BrdU/NSE-positive double-labeled cells than BrdU/GFAP-positive double-labeled cells (Figure 5). Conversely, the BMSC group had more BrdU/GFAP-positive double-labeled cells than BrdU/NSE-positive double-labeled cells (Table 3).

**Table 3:** **Effects of BMSC-Ns and BMSCs transplantation on neurological performance, BrdU labeling, and cell differentiation (data represent mean ± SD, *n* = 8 per group): comparison of the antigen differentiation ratios of neural cells after cell implantation**.

Group	NSE differentiation ratio	GFAP differentiation ratio
BMSC-N	0.57 ± 0.05	0.4 ± 0.02^a^^,^^b^
BMSC	0.12 ± 0.03	0.32 ± 0.04^b^
Control	0.00 ± 0.00	0.00 ± 0.00

*Data represent mean ± SD*.

*^a^BMSC-N and BMSC groups are significantly different (p < 0.01)*.

*^b^NSE differentiation ratio and GFAP differentiation ratio are significantly different p < 0.01*.

### Severity of Brain Injury and Brain Cell Morphology Evaluated Using HE Staining

**Figure 6:**
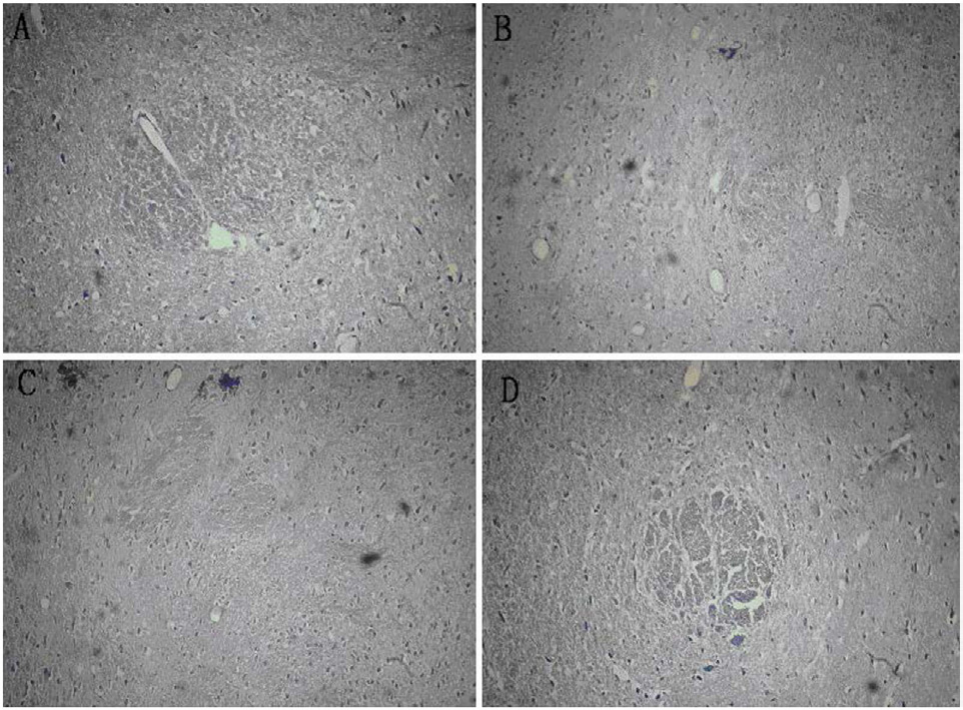
**Mensuration of infarct volumes in various groups before and after transplantation by HE staining**. **(A)** The ratio of infarct area was 0.83% after modeling, **(B)** the ratio of infarct area was 0.38% in the BMSC-N group, **(C)** the ratio of infarct area was 0.51% in the BMSC group, and **(D)** the ratio of infarct area was 0.57% in the control group.

At day 7 after MCAO, it was visible that the nerve cells were significantly reduced in size in the right frontotemporal cortex and basal ganglia; the neurons in the infarct area and the surrounding surviving neurons had shrunk and became deformed; the nucleus had shrunk and became deformed, fragmented, or completely dissolved; and the interstitial edema was significant. Under light microscope by HE staining, it was observed that all rats in three groups showed cystic necrosis in the cerebral ischemia area; a large number of neurons were lost; there were more small grid cells and few neutrophils; and the nucleus had shrunk and become deformed in the area surrounding the necrosis, where unobvious proliferation of glial cells and leukocyte infiltration appeared. The damage and loss of nerve cells in the BMSC-Ns group and BMSCs group were relatively mild. There were no hyperchromatic heterocysts with larger nuclei visible in the area surrounding the necrosis. The proliferation of glial cells in the BMSC-Ns group was less than that of the BMSCs group. There was no significant difference between various groups before transplantation; the infarct area in each group was reduced after transplantation compared with that before transplantation (*p* < 0.05) (0.37 ± 0.019%) in the BMSC-Ns group, (0.52 ± 0.048%) in the BMSCs group, and (0.57 ± 0.031%) in the control group. The infarct volume was reduced in the BMSC-Ns group compared with the control group (*p* < 0.01), but was not significantly different between the BMSCs group and the control group (Figure 6).

## Discussion

In recent years, with the development of regenerative medicine, stem cell transplantation for the treatment of central nervous system disease is considered to be a way of great potential. MSCs are considered as one of the most useful types of stem cells (15). However, studies have shown that the ratio of BMSCs differentiating into glial cells and fibroblasts after transplantation *in vivo* is higher than the ratio of BMSCs differentiating into neuron-like cells (7, 8). Excessive glia and fibroblasts can increase glial scar formation (9). We theorized that BMSCs *in vitro* differentiation into more neuronal precursor cells than glia and fibroblasts before transplantation may represent a more effective therapeutic strategy. At present, several are methods used to induce the differentiation of MSCs into neural cells. These include exposure to cytokines, such as NGF, EGF, and bFGF (16); chemical induction with substances (17); exposure to brain homogenate (18); and gene transfection (19). Cytokines are commonly used as induction agents because they play a role in promoting stem cell proliferation and differentiation through their own receptors (20). We have demonstrated that hCSF-treated human BMSCs could successfully differentiate into neuron-like cells (10, 11). Three days of hCSF-induction prompts neurite growth, and the cultured cells gradually developed into a tapered, triangular, and irregular shape. The soma on day 7 was similar to the dendrite and axon-like structures of astrocytes. Compared with cytokine-induced cells, those that were induced with hCSF were quicker to differentiate and which also occurred in higher proportions (10, 11). This has resolved numerous problems associated with the use of culture media and stimulating factors.

The hCSF contains sufficient components to induce the differentiation of MSCs into NSCs. hCSF provides a physiological microenvironment for the differentiation of MSCs into NSCs, which can not be substituted by artificial culture medium. The combination of hCSF and BMSCs presents a promise for clinical application since both the hCSF and bone marrow could be obtained from patient himself without considering immune system. Since 2009, the Chinese government has been attempting to implement a clear policy for the clinical application of stem cells (http://www.stemcellsportal.com). We have demonstrated that hCSF-induced BMSC-Ns transplantation facilitates recovery in seven patients with nervous system diseases (21), suggesting auto-BMSCs transplantation is a feasible, convenient, safe, and effective therapeutic approach. However, the survival, differentiation, and integration with the organism of hCSF-induced BMSC-Ns in the brain of live animals after transplantation remain elusive. The total volume of adult rat CSF is approximately 580 μl, which limits the collection of a sufficient volume of rat CSF. In the present study, we used hCSF to induce rat BMSCs as transplanted cells. At day 4 post-induction by hCSF, cells were positive for both NSE and GFAP, but expression levels for NSE were higher than GFAP. This result is consistent with our previous results showing that induction of human BMSCs differentiation into neuron-like Cells with hCSF (10, 11).

The current mostly used transplantation ways in the treatment of central nervous system diseases include target transplantation, peripheral blood vessel injection (vein and artery), and cerebrospinal fluid transplantation (22–24). However, there is conflict in the choosing of transplantation ways, while which one is the best way remains unknown. Intravenous transplant is a way that in which NSCs are dropped into the blood circulation system through venipuncture, by which stem cells reach the nervous system *via* blood circulation where stem cell shows its ability by penetrating blood–brain barrier. However, in addition to the long way to reach, long time to work, and the cells consumption, there are only 30% transplanted stem cells reach the central nervous system, while the rest are distributed in peripheral blood circulation. Transplantation through artery, which is associated with more cell conservation, however, is difficult to be utilized in clinical work. Ventricle puncture, through which transplanted stem cells could reach the ventricular system directly and then located the entire nervous system *via* circulation, which is also related to a higher transplant point, a shorter pass way, and a fewer loss of stem cell. Furthermore, transplantation through ventricle puncture may avoid volume mass effect and increase the number of transplanted cells, while brain environment provides a good place for migration development and promote the directed differentiation of the implanted NSCs, in that the ventricle implanted cells could quickly reach the damage zone of the brain. Furthermore, cell transplantation can be achieved by lumbar puncture and intrathecal injection, this is in line with our reported clinical work (21). Studies have shown that within 24 hours after injury, most of the transplanted cells did not survive in the injured area and the lateral ventricles, this may be a result of from pathphysiological changes and secondary injury which are not conducive to the survival of transplanted cells. We transplanted stem cells 7 days after injury when the microenvironment was improved. Additionally, it would take 7 days to collect and induce the direct differentiation of the autologous BMSCs. Stem cells have been reported to fail multi-lineage development when transplanted at ultra low density (3, 4), making assessment of dose-related integration into the niche an important variable. In this regard, while it has generally been assumed that “more is better,” in clinical trials, limited by small amount of autologous BMSCs, the number of stem cells was 0.54–2.8 × 10^8^ (24). We chose 2 × 10^6^ cells in rat in our experiment accordingly.

Our data revealed that hCSF-induced rat BMSC-Ns transplantation in the lateral ventricles of MCAO rats significantly improved behavioral performances and cell differentiation as compared with BMSC group. We also found that BrdU+ cells were more than 80% when the cell confluence was over 90%. The BrdU+ cells can be observed even 32 days after transplantation. The number of BrdU+ cells in the brain tissue after transplantation of hCSF-induced BMSC-Ns was significantly higher than that of hCSF-induced BMSCs. The numbers of both BrdU/GFAP-positive double-labeled and BrdU/NSE-positive double-labeled cells in the BMSC-N group were significantly higher than those in the BMSC group. However, the number of BrdU/NSE-positive double-labeled cells was greater than that of the BrdU/GFAP-positive double-labeled cells in the BMSC-N group and *vice versa*. These results indicate that the BMSCs survived *in vivo* after transplantation tend to differentiate into astrocyte-like cells and that more neuron-like cells were found in the BMSC-N group than in the BMSC group. The *in vivo* migration and differentiation of BMSCs in the present study were consistent with reports from other laboratories (25, 26). This result also supports our hypothesis that induction and differentiation of BMSCs toward specific target cells according to clinical purposes before transplantation may be a more effective treatment strategy than transplanting BMSCs prior to differentiation.

In this study, we found that neurological function was significantly improved 4 days after transplantation of eitherBMSCs or BMSC-Ns. This improvement could not be attributed to that necrotic cells were replaced by transplanted cells. Previous studies have demonstrated that the secretion, rather than substitution of local cells, during the early stage of implanted stem cell play the dominant role (7, 8, 27). The cells surrounding the early cerebral ischemic area are fragile and prone to apoptosis. Therefore, it is essential to protect the cells surrounding the damaged brain from being harmed by apoptosis. BMSCs and BMSC-Ns could enter the brain tissue and promote self-healing of neural tissue and improve neurological function through their own synthesis and secretion (28). They could also promote release of neurotrophic factor, vascular endothelial growth factor, nerve growth factor, and stem cell growth factor, which increase survival, migration, directed differentiation into neurons of endogenous NSCs, and injury repairmen (29, 30). The *in vitro* differentiation rate of BMSCs into neuron-like cells can be as high as 80%. However, in pathological condition, the *in vivo* conversion rate of BMSCs is only 3–10% regardless of the methodology of transplantation into the cerebral ischemia area (31–33). After transplantation, the ratio of BMSCs differentiating into glial cells and fibroblasts is higher than the ratio of BMSCs differentiation into neuron-like cells *in vivo*. Excessive glia and fibroblasts potentiate the formation of glial scars, which restricts the growth of the axon in the damaged area or the recovery of neurological function. There is no doubt that glial scar formation impedes and negatively affects the recovery of nervous system function. Therefore, many researchers believe that it would be difficult to reconstruct neural pathways by completely replacing damaged nerve tissue using *in vivo* differentiation of BMSCs. After transplantation *in vivo* in the present study, differentiation of hCSF-induced BMSC-Ns into neuron-like cells was significantly higher, with less differentiation into glia-like cells than that of BMSCs. The early cellular secretion could support and provide nutrition to the local nerve tissues, and thus promote cell repair and functional recovery. However, in our research, neurological function significantly improved later. Our results indicate that the replacement of damaged or necrotic neurons by BMSC-Ns for reestablishment of neural pathways is a feasible therapeutic approach in the recovery of nervous system functions.

## Author Contributions

This work has not been published previously and does not conflict with any actual and potential interests. All authors listed have contributed to the manuscript, both in the data collection and article preparation. All authors have approved the publication of the final manuscript. Corresponding author: TX, M.D. YY and Y-rP are both first author.

## Conflict of Interest Statement

The authors declare that the research was conducted in the absence of any commercial or financial relationships that could be construed as a potential conflict of interest.
