# Supplementary material for: In Vitro Differentiation of Bone Marrow Mesenchymal Stem Cells into Neuron-Like Cells by Cerebrospinal Fluid Improves Motor Function of Middle Cerebral Artery Occlusion Rats
Source: Front Neurol. 2016 Oct 27;7:183. doi: 10.3389/fneur.2016.00183 (PMC5081354; doi:10.3389/fneur.2016.00183)
Supplement: Supplementary file 1 [file Table_1.DOC]

Modified NSS score

**Items Scores**

| **Motor tests** |  |
| --- | --- |
| **Raising rat by the tail** | **3** |
| Flexion of forelimb | 1 |
| Flexion of hindlimb | 1 |
| Head moved >10° to vertical axis within 30 s | 1 |
| **Placing rat on the floor (normal=0; maximum=3)** | **3** |
| Normal walk | 0 |
| Inability to walk straight | 1 |
| Circling toward the paretic side | 2 |
| Fall down to the paretic side | 3 |
| **Sensory tests** | **2** |
| Placing test (visual and tactile test) | 1 |
| Proprioceptive test (deep sensation, pushing the paw against the table edge to stimulate limb muscles) | 2 |
| **Beam balance tests (normal=0; maximum=6)** | **6** |
| Balances with steady posture | 0 |
| Grasps side of beam | 1 |
| Hugs the beam and one limb falls down from the beam | 2 |
| Hugs the beam and two limbs fall down from the beam, or spins on beam (>60 s) | 3 |
| Attempts to balance on the beam but falls off (>40 s) | 4 |
| Attempts to balance on the beam but falls off (>20 s) | 5 |
| Falls off: No attempt to balance or hang on to the beam (<20 s) | 6 |
| **Reflexes absent and abnormal movements** | **4** |
| Pinna reflex (head shake when touching the auditory meatus) | 1 |
| Corneal reflex (eye blink when lightly touching the cornea with cotton) | 1 |
| Startle reflex (motor response to a brief noise from snapping a clipboard paper) | 1 |
| Seizures, myoclonus, myodystony | 1 |
| **Maximum points** | **18** |
